# Deciphering structure-composition-efficacy transformation in processed Fuzi: an integrated SEM, XRD, metabolomics, and pharmacodynamics approach

**DOI:** 10.3389/fchem.2025.1630159

**Published:** 2025-09-11

**Authors:** Yang Yang, Xuemei Wei, Shuya Zhang, Yun Li, Jiazhen Wu, Suhua Cai, Biren Wang, Zhiming Yang, E. Yang, Junling Cao, Qinhua Chen, Guihong Chen

**Affiliations:** 1 Shenzhen Baoan Authentic TCM Therapy Hospital, Shenzhen, China; 2 Shenzhen Bao’an Chinese Medicine Hospital, Guangzhou University of Chinese Medicine, Shenzhen, China

**Keywords:** Fuzi, TCM processing standardization, starch ultrastructure, metabolomics, efficacy-safety balance

## Abstract

**Background:**

*Aconiti Lateralis Radix Praeparata* (Fuzi), a vital yet toxic herbal medicine, faces significant challenges in standardization due to varied processing methods that undermine detoxification efficacy and hinder therapeutic optimization.

**Methods:**

An integrative analytical approach combining scanning electron microscopy (SEM), X-ray diffraction (XRD), metabolomics, and pharmacodynamic evaluation was employed to investigate the structural, compositional, and functional transformations during Fuzi processing.

**Results:**

SEM imaging and XRD data revealed that the morphological breakdown of starch granules varied according to processing technique. Metabolomics analysis based on mass spectrum identified 57 diterpenoid alkaloids, including 12 bioactive components, whose levels fluctuated across nine processing variants. Pharmacodynamic profiling revealed distinct efficacy-safety trade-offs across these variants, underscoring the pivotal role of processing methods in determining clinical risk profiles and therapeutic indices.

**Conclusion:**

This study elucidated the structure-composition-efficacy correlations in Fuzi processing, highlighting the relationship between starch reorganization, alkaloid modification, and therapeutic outcomes. These insights offer a mechanistic basis for refining traditional processing protocols, optimizing detoxification and therapeutic retention, and advancing standardization in herbal medicine.

## 1 Introduction

*Aconiti Lateralis Radix Praeparata* (Fuzi), derived from the processed lateral roots of Aconitum carmichaelii Debx, demonstrates a wide range of pharmacological properties, including cardiotonic, antiarrhythmic, anti-inflammatory, analgesic, and antitumor effects, but also exhibits toxicities due to its diterpenoid alkaloid (DA) constituents (Zhou et al., 2015). Although the 2020 Chinese Pharmacopoeia recognizes four approved processed forms (Hei shun pian, Bai fu pian, Pao fu pian, Dan fu pian) for clinical use, over ten regional variants exist, resulting from inconsistent processing methods (Chinese Pharmacopoeia Commission, 2020). This variability complicates the understanding of detoxification mechanisms and modulation of active components, presenting challenges for quality control (QC) and clinical safety. Developing robust analytical frameworks to monitor the dynamic changes in alkaloid subtypes during processing is essential for standardizing Fuzi’s safety and efficacy.

Traditional Chinese “Pao zhi” techniques, such as steaming and boiling, convert the toxic aconitine alkaloids in Fuzi into less harmful compounds, thereby reducing toxicity while preserving therapeutic efficacy (Lei et al., 2021; Yang et al., 2018). However, the role of starch, a key component undergoing significant physicochemical transformations during processing, remains inadequately understood in terms of its influence on quality, stability, and clinical safety (Liu et al., 2017; Xia et al., 2011). Clarifying the dynamics of starch during processing is vital for optimizing Pao zhi protocols and enhancing the evidence-based, safe application of this high-risk herbal medicine (Ye et al., 2019). Different processing methods yield distinct pharmacological profiles: Dan Fu Pian alleviates depression and pain, Pao Fu Pian addresses Yang deficiency through kidney and spleen warming (Wang, 2014), Chao Fu Pian excels in anti-inflammatory effects (Fan et al., 2020), while Hei Shun Pian demonstrates immunosuppressive properties, highlighting how processing-induced chemical remodelling underpins the clinical diversity of Fuzi’s applications (Xiong et al., 2017).

**FIGURE 1:**
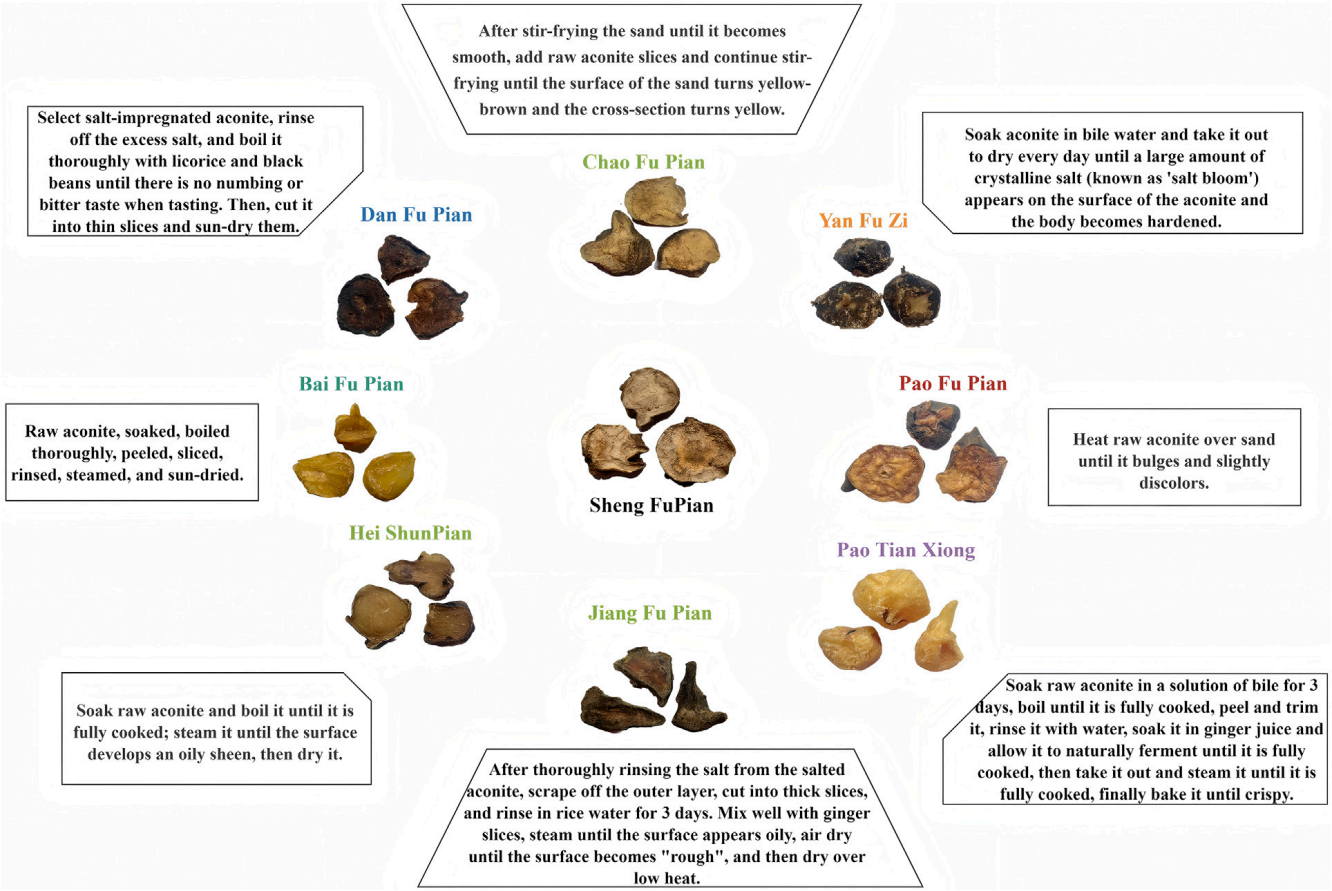
Description and appearance of nine types of Fuzi and their processed products.

This study investigated the relationship between detoxification and component retention through electron microscopy characteristics and processing variations, aiming to establish a physical basis for restoring the efficacy of traditional processed Fuzi products. By employing metabolomics analysis, the degradation pathways of toxic components and retention levels of active ingredients across nine different processing methods (Figure 1) were elucidated. This analysis identified processing methods that reduce toxicity while preserving essential medicinal properties, offering a chemical basis for the development of standardized protocols that balance toxicity reduction with efficacy preservation. The evaluation of pharmacological differences across processed products will help guide clinical decision-making regarding the selection of appropriate forms, ensuring a balance between efficacy and safety. This multidimensional approach strengthens the scientific foundation of herbal processing theory, providing comprehensive strategies to optimize Fuzi processing methods, clarify pharmacologically active constituents, and offer critical insights for clinical use with balanced efficacy and safety profiles. These findings will facilitate the standardization and optimization of processing techniques for Aconitum, ultimately enhancing its safe and effective application in clinical settings.

## 2 Materials and methods

### 2.1 Sample and chemicals

ShengFuzi, derived from the lateral roots of *Aconitum carmichaelii* Debx, was provided by Kangmei Pharmaceutical Co., Ltd. (Chengdu, China) in May 2023. The authentic sample (Fuzi KM 202305) is stored at the Shenzhen Bao’an Authentic TCM Therapy Hospital, Shenzhen, Guangdong, China. LC-MS-grade methanol (MeOH), formic acid (FA), acetonitrile, and isopropanol were sourced from Sigma-Aldrich (United States). HPLC-grade ammonium acetate was obtained from Chengdu Chron Chemical Co., Ltd. (Chengdu, China). Purified water for UPLC analyses was produced using the Elga Labwater Purelab system (Elga-Veolia, High Wycombe, United Kingdom). All other reagents were of analytical grade. MeOH of analytical grade for sample preparation was procured from Shanghai Chemical Reagent Company (China Pharmaceutical Group). Chromatographic-grade MeOH, acetonitrile, and isopropanol for UPLC and LC were obtained from Merck KGaA (Darmstadt, Germany). The mobile phase additives, ammonia and FA, were mass spectrometry grade and supplied by Shanghai Aladdin Biochemical Technology Co., Ltd. (Shanghai, China).

The reference substances for the 12 processed aconite products include aconine, mesaconine, hypaconine, hypaconitine, mesaconitine, lappaconitine, benzoylaconine, benzoylmesaconine, benzoylhypaconine, karacoline, fuziline, and songorine, with Yohimbine used as the internal standard (IS). A detailed list of these reference substances is provided in Supplementary Table S1.

### 2.2 Instrumentation and equipment

Analytical instrumentation included the TESCAN MIRA3 field emission scanning electron microscope, gold sprayer, energy spectrometer, and an electronic analytical balance (Mettler Toledo Instruments Co., Ltd.); injection pump (Baoding Rafer Fluid Technology Co., Ltd.); Sartorius Pure Water Machine (Sartorius (Shanghai) Trading Co., Ltd.); H-Class UPLC SynaptG2 Si QTOF; DA type CNC ultrasonic cleaner (Dongguan Keqiao Ultrasonic Equipment Co., Ltd.); DP-01 oil-free vacuum pump (Shanghai Andar Industrial Co., Ltd.); R300 rotary evaporator (Buqi Co., Ltd., Switzerland); Waters Xevo G2-S Q-TOF mass spectrometer (Waters, United States); P180H ultrasonic water bath (Elma, Germany); Quintix 224-1 CN-1 Electronic Balance and Quintix 125-1 CN Electronic Balance (Sedolis Beijing Co., Ltd.); UHPLC/LTQ Orbitrap analysis was performed using HPLC coupled with Thermo linear ion trap electrostatic field orbital trap (LTQ Orbitrap) Velos Pro mass spectrometry (Thermo Fisher Scientific, San Jose, CA, United States), with Xcalibur 2.1 software for instrument control and data processing.

### 2.3 Processing procedure

The aconite products in this study were processed according to the method outlined in Supplementary Table S2, under the supervision of Professor Mei Quanxi and executed by the processing studio of Jin Shiyuan, a renowned master of traditional Chinese medicine in Shenzhen, Guangdong Province. Detailed characteristics of the processed aconite products are presented in Figure 1. This precise processing procedure ensured consistency and quality, critical for the reproducibility and reliability of experimental outcomes. All experimental protocols used a single batch of raw Aconiti Radix as the starting material. Uniform processing was carried out by a designated researcher following the protocols detailed in Supplementary Table S2, with three independent replicates for each processed product to ensure reproducibility.

### 2.4 Scanning electron microscopy (SEM) analysis

#### 2.4.1 Physicochemical characterization of starch

Starch content was quantified using the AOAC Official Method 996.11. The amylose content in Fuzi-derived starch was determined via iodine colorimetry, following the procedure outlined by Castro et al. (2023). Swelling power and aqueous solubility were assessed simultaneously, based on the protocol by Kaul et al. (2023), while water-binding capacity (WBC) was measured according to Wang M. et al. (2023). All analyses were performed in triplicate to ensure methodological rigor, and results were normalized to dry starch weight for cross-comparison.

#### 2.4.2 SEM

The morphological characteristics of the starch samples were analyzed using an Inspect F50 scanning electron microscope (FEI, Hillsboro, United States). Imaging was conducted at an accelerating voltage of 10 kV and a magnification of 2,000×, enabling detailed visualization of the surface structure and particle morphology of the starch samples (Yang et al., 2019).

#### 2.4.3 X-ray diffraction (XRD)

XRD analysis was performed to examine the crystalline structure of the starch samples. XRD patterns were recorded using a D8 ADVANCE diffractometer (Bruker, Karlsruhe, Germany) with Ni-filtered Cu-Kα radiation (40 kV, 30 mA). The analysis was conducted under the following conditions: diffraction angle (2θ) range from 4° to 60°, scanning speed of 3°/min, and a step size of 0.02°. The relative crystallinity of the samples was calculated based on the method described by Wang C. et al. (2023).

#### 2.4.4 Statistical analysis

All measurements were performed in triplicate to ensure result reliability and reproducibility. Statistical analysis was carried out using one-way ANOVA, followed by Duncan’s multiple range test for pairwise comparisons of means. Statistical evaluations were conducted using SPSS 19.0 (SPSS, Chicago, United States), with differences considered statistically significant at P < 0.05 (Wang et al., 2024).

### 2.5 Analysis of diterpenoid alkaloids in different processed products of aconite

#### 2.5.1 Sample preparation

Reference Solution Preparation: Accurate amounts of 12 reference substances were weighed and dissolved in 75% MeOH containing 0.025% acetic acid to prepare individual stock solutions. These stock solutions were then diluted with the same solvent to create 11 mixed solutions with varying concentration gradients, which were used to construct standard curves for the quantification of DAs.

Test Solution Preparation: Approximately 2.0 g of processed aconite powder (sieved through a No. 3 sieve) was accurately weighed and transferred to a 125 mL conical flask. Thirty milliliters of 75% MeOH solution (containing 0.025% acetic acid) was added, and the mixture was subjected to ultrasonic treatment for 30 min. The solution was then filtered, and the residue was washed with an additional 30 mL of 75% MeOH solution, which was also treated ultrasonically for 30 min and subsequently filtered. The combined filtrates were concentrated under reduced pressure, re-dissolved in 10 mL of 75% MeOH solution (containing 0.025% acetic acid), and centrifuged at 14,000 rpm for 10 min. The supernatant was collected, and 390 µL of the supernatant was mixed with 10 µL of an IS solution to prepare the test solution.

QC Sample Solution Preparation: Equal volumes of each group of test sample solutions were combined to prepare the QC sample solution. Three hundred microliters of the QC sample solution was evaporated under a nitrogen stream at 40 °C and re-dissolved in 100 µL of the same solvent (including the IS) to obtain a threefold concentrated QC sample solution. These precise sample preparation steps ensure the accuracy and reliability of subsequent analyses, providing a solid foundation for the quantification and characterization of DAs in various processed aconite products.

#### 2.5.2 UHPLC/LTQ orbitrap mass spectrometry analysis method

Ultra-high performance liquid chromatography (UHPLC) coupled with linear trap quadrupole (LTQ) Orbitrap mass spectrometry (MS) was performed using a Thermo Ultimate 3000 UHPLC system fitted with a Waters CSH C18 column (2.1 × 100 mm, 1.7 μm particle size). The column was maintained at 30 °C, with the mobile phase consisting of 0.1% FA in water (A) and acetonitrile (B). The gradient elution program was as follows: 0–4°min, 5%–8% B; 4–10°min, 8%–18% B; 10–15 min, 18%–30% B; 15–20°min, 30%–40% B; 20–25°min, 40%–100% B; 25–30°min, 100% B. The flow rate was set to 0.3 mL/min, and the injection volume was 1 μL. Mass spectrometry analysis was conducted using an LTQ Orbitrap Velos Pro mass spectrometer in positive ion mode. The electrospray ionization (ESI) source parameters were as follows: source voltage 3.8 kV, capillary temperature 350 °C, source heater temperature 300 °C, sheath gas (nitrogen) flow rate 40 arbitrary units, auxiliary gas (nitrogen) flow rate 10 arbitrary units, and sweep gas (nitrogen) flow rate 2 arbitrary units. Collision-induced dissociation (CID) was employed in multi-stage mass spectrometry (MS/MS). Event 1 comprised a first-order full scan with a mass range of m/z 100–1,000, a resolution of 30,000, and data acquisition in profile mode. Events 2 and 3 initiated CID fragmentation of the most abundant ions from events 1 and 2, respectively, with a normalized collision energy (NCE) of 35%, scanning resolution of 7,500, and data recorded in centroid mode. Dynamic exclusion parameters were configured as follows: repeat count = 2, repeat duration = 10 s, exclusion list size = 50, exclusion duration = 10 s, and low and high exclusion mass width = 1.5 Da. Data acquisition and processing were performed using Xcalibur 2.1 software, with the following atom settings: C 0–60, H 0–100, O 0–20, N 0–1, unsaturation 5–25, and mass deviation <10 ppm. This precise and comprehensive approach ensures accurate quantification and characterization of DAs in various processed aconite products.

#### 2.5.3 HPLC-MS/MS analysis method

The HPLC-MS/MS system consisted of an HPLC separation system (Shimadzu, Kyoto, Japan) coupled with an API 6500+ Qtrap mass spectrometer, equipped with an ESI interface (AB Sciex, Framingham, MA, United States). Chromatographic separation was performed using an ACQUITY UPLC BEH C18 column (2.1°mm × 50 mm, 1.7°μm, Waters Corp., Milford, MA, United States) with the column temperature maintained at 40 °C. The mobile phase consisted of 0.1% FA in water (phase A) and acetonitrile (phase B). A gradient elution program was employed at a flow rate of 0.4 mL/min, as follows: 0–1°min, 10% B; 1–4°min, 10%–90% B; 4–8°min, 90% B; 8–9°min, 90%–10%; 9–10°min, 10% B. In positive ion mode of the ESI source, multiple reaction monitoring (MRM) acquisition was performed with the following settings: spray voltage at 5,500 V, curtain gas (CUR) at 35°psi, collision gas (CAD) set to medium, ion source temperature (TEM) at 550 °C, ion source gas flow 1 (GS1) at 55°psi, and ion source gas flow 2 (GS2) at 55°psi.

#### 2.5.4 Establishment of a diterpene alkaloid database for aconitum

To systematically catalog the chemical constituents of *Aconitum carmichaelii Debx*, a comprehensive literature search on the extraction and separation of aconite was conducted, supplemented by data from databases such as SciFinder. By 2023, a database of 160 DAs isolated from *Aconitum carmichaelii* Debx was compiled. This database includes 121 C19 DAs and 39 C20 DAs, with detailed records for each compound, including name, molecular formula, molecular weight, CAS number, and chemical structure. Using the Compound Discoverer 3.1 software platform, a robust database of known DAs was constructed, serving as a valuable resource for guiding compound separation and facilitating rapid retrieval and analysis of chemical components through liquid chromatography-mass spectrometry (LC-MS) technology.

#### 2.5.5 Establishment of multi-component quantitative analysis method

To accurately determine and compare the contents of various DAs in different processed products of *Radix Aconiti Lateralis Preparata*, ultra-performance liquid chromatography coupled with quadrupole-time-of-flight mass spectrometry (UPLC/Q-TOF) analysis was performed. This analysis was benchmarked against known structurally identified compounds. By leveraging the mass spectrometric fragmentation patterns and fragment ions of DAs with distinct structural subtypes, 12 DAs with high concentrations across various processed products were identified and selected. These alkaloids, representing different structural subtypes and confirmed structures, were used to establish a multi-component quantitative analysis method.

To optimize the mass spectrometry parameters for the quantitative ion pairs of the 12 reference solutions in positive ion mode, MassHunter Optimizer software was employed. The optimization process involved several steps: setting the range of fragment and collision energy (CE) values, parent ion identification, fragment value optimization, sub-ion selection, and final CE value optimization. In MassHunter Optimizer software, the fragment value range was set to 100–300 V, and the CE value was set between 10-60 V. Parent ions were identified through data acquisition in MS2 scan mode. The fragment value was optimized in MS2 selected ion monitoring (SIM) mode to ensure efficient transmission of parent ions. Sub-ions were selected based on high-intensity signals during product ion scanning mode, using the optimized fragment value for data collection. The CE value was further fine-tuned in MRM mode to maximize sub-ion response. The optimized fragment and CE values were then applied to the quantitative analysis.

Under these optimized conditions, both parent ions and sub-ions were simultaneously monitored, ensuring high sensitivity and selectivity. The main mass spectrometry parameters after optimization are summarized in Supplementary Table S3. This approach guarantees accurate and reliable quantification of multiple DAs, supporting comprehensive QC and facilitating comparative analysis of different processed products of *Radix Aconiti Lateralis Preparata*.

### 2.6 Methodological validation

#### 2.6.1 Specificity and linearity

The specificity of the method was verified by comparing the base peak chromatograms (BPC) of the blank solvent, sample solution, and reference mixture under dynamic MRM mode. As shown in Supplementary Figure S1, no interfering substances matching the retention times of the 12 reference compounds were detected in the blank solvent. However, components corresponding to the retention times of the reference compounds were observed in the sample solution, confirming the method’s good specificity. Linearity was assessed by injecting 11 reference mixtures with varying concentration gradients. A standard curve was constructed by plotting the relative response of the analyte against the IS on the y-axis and the relative concentration on the x-axis. The limit of quantification (LOQ) was established based on a signal-to-noise ratio of at least 10. The regression equations and linear range for each component are summarized in Supplementary Table S4. This rigorous validation ensures the method’s specificity and linearity, enabling reliable and accurate quantification of the target compounds.

#### 2.6.2 Reproducibility and precision

The method’s reproducibility and precision were evaluated through a series of tests on Heishun tablets. Repeatability was assessed by calculating the relative standard deviation (RSD) of the solution content from six parallel samples of Heishun tablets. Intra-day precision was determined by injecting the test solution six times consecutively and calculating the RSD of the content. Inter-day precision was evaluated by preparing six parallel samples of Heishun tablets and injecting them over three consecutive days, followed by calculation of the RSD. The results, summarized in Supplementary Table S5, suggest that the RSD for repeatability ranged from 1.09% to 9.40%. The intra-day RSD ranged from 1.67% to 8.62%, while the inter-day RSD ranged from 2.82% to 9.78%. These results demonstrate the method’s excellent repeatability and precision, ensuring reliable and consistent quantification of the active ingredients in Heishun tablets. The low RSD values in both intra-day and inter-day precision tests confirm the method’s robustness, which is essential for QC and regulatory compliance.

#### 2.6.3 Recovery rate and stability

The method’s accuracy was evaluated by assessing the recovery rates of six parallel samples at medium concentrations. Recovery rates were determined by spiking known amounts of the analyte into the samples and comparing the measured concentrations with the expected values. The results, summarized in Supplementary Table S6, confirm the method’s accuracy in quantifying the analyte within the matrix, validating the reliability of the analytical procedure. To assess the stability of the test solution, it was placed in an automatic sampler at room temperature for 0–24 h, with injections every 4 h. Stability was evaluated by calculating the RSD of the peak areas between the compound under test and the IS. The results in Supplementary Table S6 indicate RSD values ranging from 1.52% to 9.45%, demonstrating excellent stability of the sample over the 24-h period and confirming that the test solution remains consistent and reliable under the specified conditions.

### 2.7 Study on the anti-cancer effect of different aconite preparations

#### 2.7.1 Cell culture

SiHA and A549 cells were obtained from Procell (Hubei, China). A549 cells were cultured in F-12K medium (MeisenCTCC, China), and SiHA cells were cultured in DMEM (GIBCO, United States). Both media were supplemented with 10% fetal bovine serum (FBS), 100 mg/mL streptomycin, and 100 U/mL penicillin (GIBCO, United States). The cells were incubated in a humidified chamber (Thermo Scientific) at 37 °C with 5% CO_2_.

#### 2.7.2 Preparation of lyophilized powder of aconite and its processed products

Four types of processed Aconiti Lateralis Radix Praeparata (Sheng Fu Pian, Dan Fu Pian, Pao Fu Pian, Hei Shun Pian) were each powdered (5 g) and transferred into 10 mL EP tubes. MeOH (10 mL) was added to each tube, followed by 30 min of ultrasonication. The mixtures were then centrifuged at 30,000 × g and 4 °C for 15 min. Supernatants were collected and lyophilized to yield product-specific freeze-dried powders. Each freeze-dried powder (0.5 g) was reconstituted in the corresponding culture medium to a final volume of 20 mL and thoroughly vortexed. The solutions were centrifuged (30,000 × g, 4 °C, 15 min), and supernatants were collected. A secondary centrifugation step was performed under the same conditions. The resulting supernatants were sterile-filtered through 0.22 μm membranes, producing final extracts with a concentration of 25 mg/mL.

#### 2.7.3 Cell viability assay

The concentrations of Sheng Fu Pian, Dan Fu Pian, Pao Fu Pian, and Hei Shun Pian used in the cell viability assay were 0, 2.5, 5, 10, 15, 20, and 25 mg/mL, respectively. Cells were treated with these concentrations for 24 and 48 h. After treatment, cells were plated in a 96-well plate, and the CCK-8 assay (Target Mol, Boston, MA, United States) was conducted following the manufacturer’s instructions. Absorbance was measured at 450 nm using a VANTAstar microplate reader (BMG Labtech). Each biological replicate included three technical replicates. Statistical analysis was performed across biological replicates (n = 3), not technical replicates. These procedures ensure a rigorous evaluation of the anti-tumor effects of various aconite preparations, providing valuable insights into their potential therapeutic applications. The cell viability assay is a key step in assessing the cytotoxicity and efficacy of these natural products, which will inform further preclinical and clinical studies.

## 3 Results

### 3.1 SEM reveals processing-driven ultrastructural dynamics in aconitum herbs: decoding detoxification-efficacy balance through physicochemical reorganization

**FIGURE 2:**
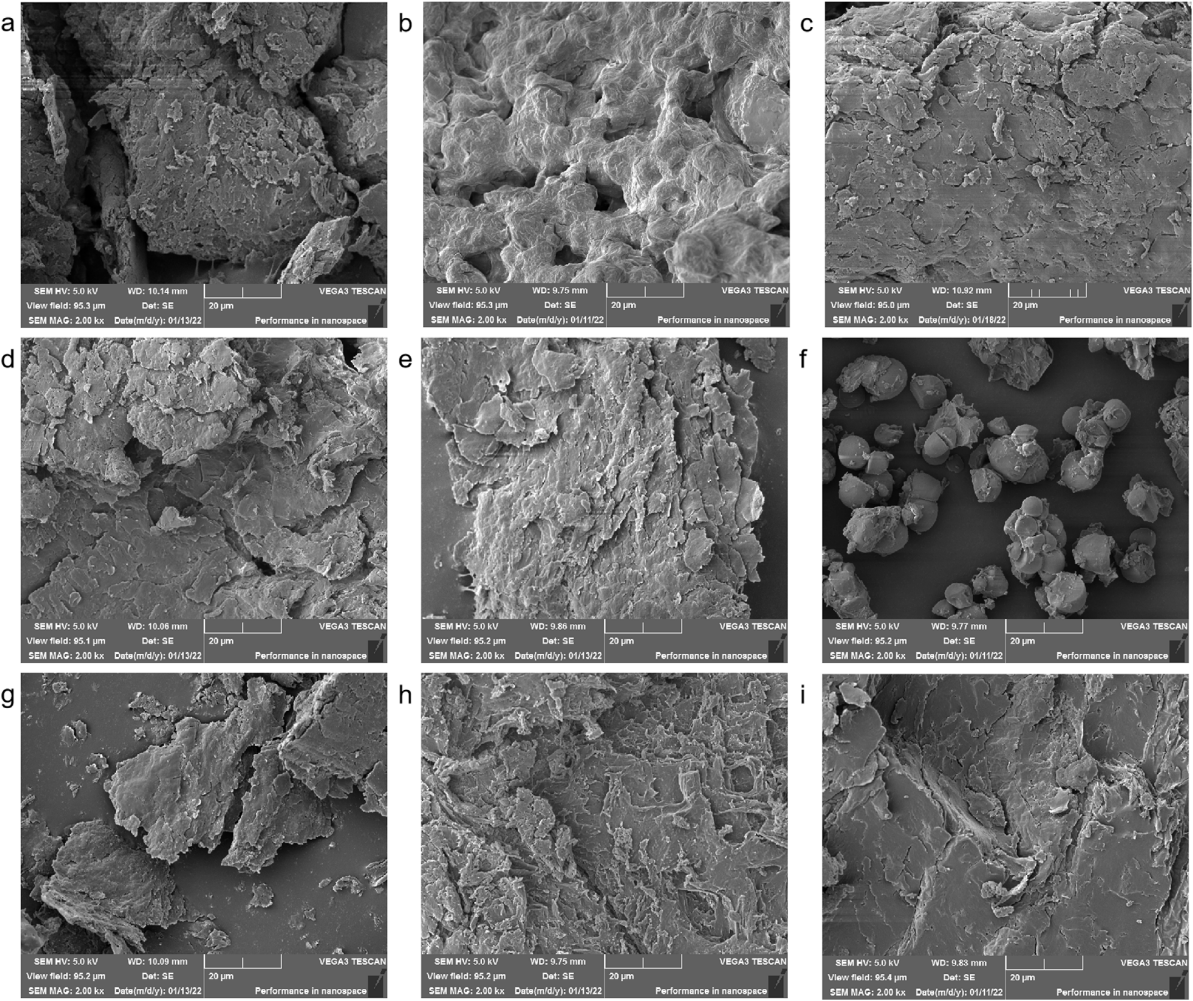
Electron microscope images of various processed products **(a)** Sheng Fu Pian, **(b)** Yan Fu Zi, **(c)** Dan Fu Pian, **(d)** Bai Fu Pian, **(e)** Hei Shun Pian, **(f)** Chao Fu Pian, **(g)** Pao Fu Pian, **(h)** Jiang Fu Pian, **(i)** Pao Tian Xiong.

SEM analysis revealed significant morphological differences in starch granules of Aconitum carmichaelii processed using distinct methods (Figure 2). During Danba processing, fresh roots were immersed in a brine solution (≥25% calcium chloride) for 7 days, followed by boiling for 20 min. This treatment caused the starch granules to expand and aggregate into amorphous lumps, likely due to gelatinization and structural disruption during soaking and thermal processing. After the roots were sliced and rinsed, the resulting starch particles were dense and irregularly shaped, indicative of aging and recrystallization induced by prolonged hydration and desalination. Further processing steps, such as steaming at 120 °C for 40 min or direct steaming followed by sun/oven drying, transformed the starch granules into irregular, flaky structures. This morphological shift aligns with gelatinization and dehydration-driven structural reorganization during high-temperature processing. These observations collectively demonstrate that processing methods induce complete disintegration of the native starch crystal structure, with SEM serving as a robust tool for differentiating processed Aconitum carmichaelii products and evaluating processing efficacy.

Supplementary Table S7 highlights a significant correlation between starch structural changes and processing techniques. Thermal processing methods such as stir-frying and roasting, applied to products like processed Fu slices, significantly elevate starch gelatinization and amylose leaching, while decreasing total starch content—likely due to caramelization and/or pyrolytic degradation during treatment. These products also exhibit substantially reduced swelling power and solubility. In contrast, moist-heat processing, such as steaming, used in preparations like Bai Fu Pian and Hei Shun Pian, enhances starch granule expansion, leading to increased swelling power and solubility compared to the raw material. Furthermore, processed Aconiti Lateralis Radix products universally show reduced protein contents, which may result from thermal denaturation and hydrolysis during processing. This reduction in protein correlates with weakened starch-protein interactions and decreased WBC.

**FIGURE 3:**
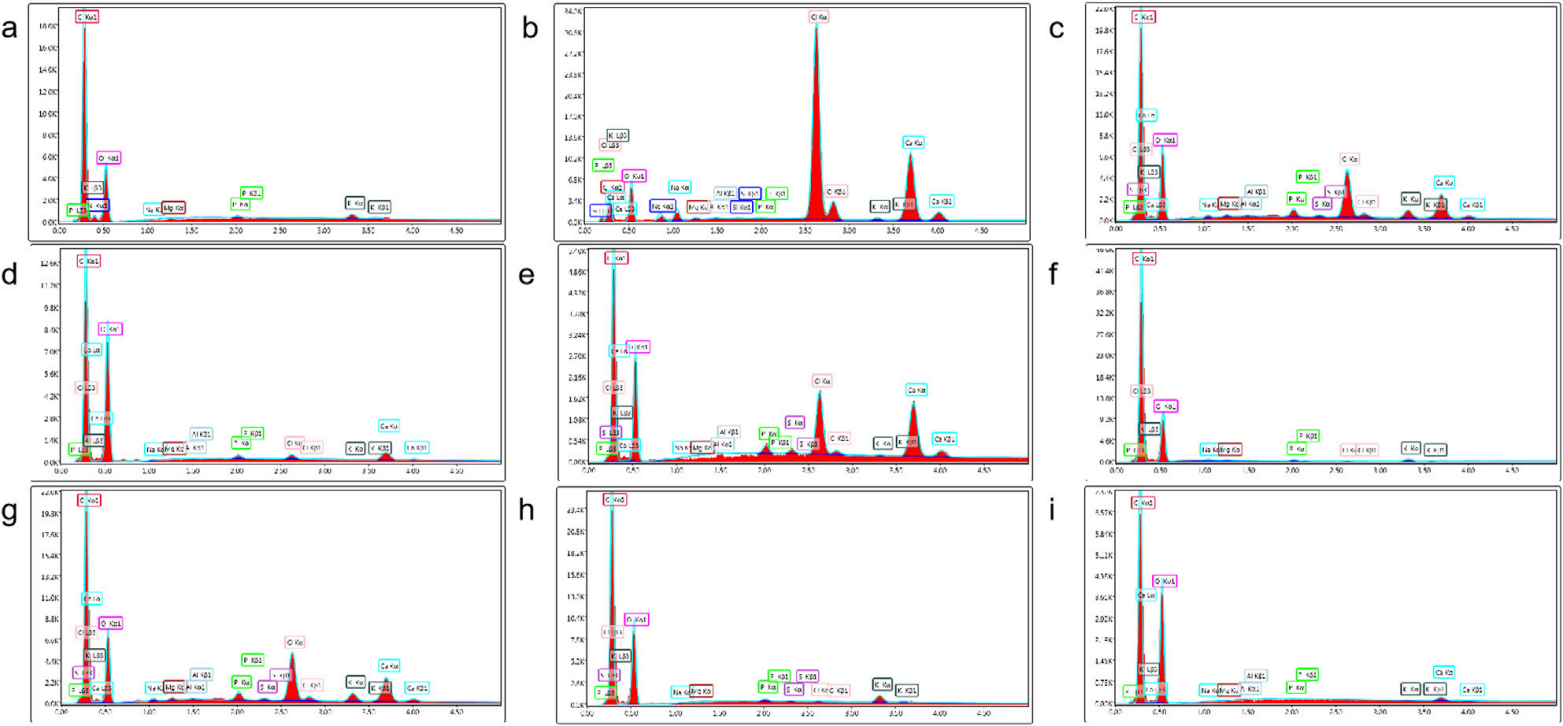
Energy spectrum analysis diagrams of various processed products. **(a)** Sheng Fu Pian, **(b)** Yan Fu Zi, **(c)** Dan Fu Pian, **(d)** Bai Fu Pian, **(e)** Hei Shun Pian, **(f)** Chao Fu Pian, **(g)** Pao Fu Pian, **(h)** Jiang Fu Pian, **(i)** Pao Tian Xiong.

XRD analysis further supported these findings. Danba processing significantly reduced diffraction peak intensity, indicating crystalline lattice disruption and gelatinization during soaking and boiling (Figure 3). In contrast, prolonged saline soaking and rinsing increased diffraction peak intensity, consistent with starch aging and recrystallization. The combination of SEM and energy dispersive spectrometry (EDS) enables rapid identification of processed products, highlighting its utility in QC and product differentiation.

Notably, specimens processed with auxiliary substances (e.g., Sheng Fu Pian) exhibit clear differences from those without supplements (e.g., Yan Fu Zi, Hei Shun Pian), which directly correlate with variations in reagent profiles. This raises significant concerns about current processing standards and underscores the urgent need for the development of safer, pharmacologically optimized methods. The findings highlight that processing-induced changes in starch structure not only modify morphological characteristics but also introduce potential chemical risks, necessitating the establishment of rigorous quality assessment frameworks. This study provides mechanistic insights into the structural dynamics of starch during Aconitum carmichaelii processing, offering a basis for enhancing product safety and standardization in traditional medicine.

### 3.2 Integrated multi-omics deciphers diterpenoid alkaloid remodeling in processed aconite: sulfonation-hydrolysis crosstalk directs precision detoxification

**FIGURE 4:**
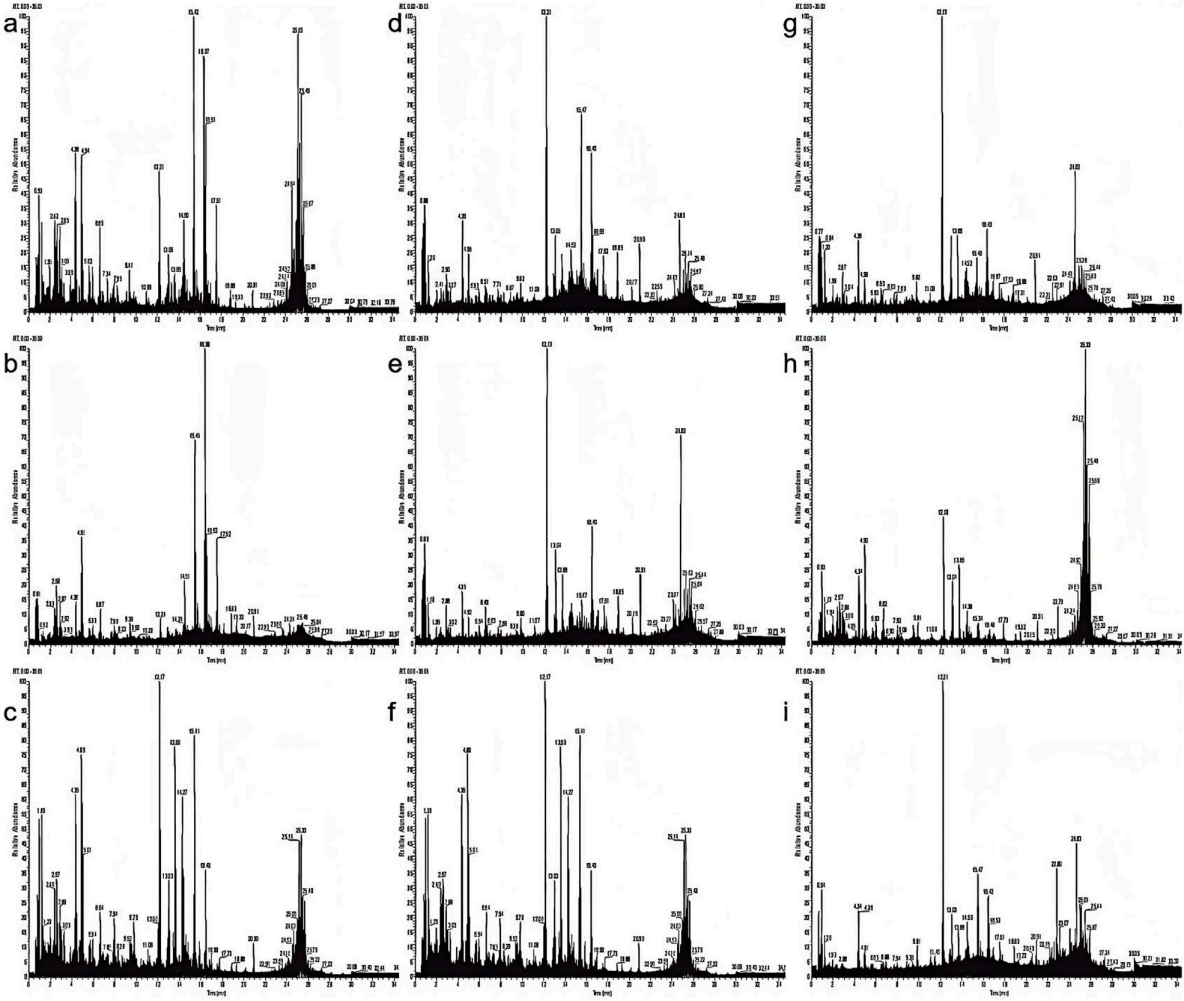
Total ion chromatograms of various processed aconite products in positive ion mode. **(a)** Sheng Fu Pian, **(b)** Yan Fu Zi, **(c)** Dan Fu Pian, **(d)** Bai Fu Pian, **(e)** Hei Shun Pian, **(f)** Chao Fu Pian, **(g)** Pao Fu Pian, **(h)** Jiang Fu Pian, **(i)** Pao Tian Xiong.

The total ion flow diagram obtained using UPLC-Q-TOF-MS/MS in positive ion mode is shown in Figure 4. The integration of LC-MS with advanced data processing workflows enables rapid and systematic profiling of DAs across various processed Aconitum carmichaelii (aconite) products, marking a critical step in understanding their structural diversity and pharmacological significance. Building on previous methodologies (Yu et al., 2022), this study employs a four-tiered analytical approach combining elemental composition analysis, degree of unsaturation (RDB) profiling, diagnostic ion filtering (DIF), neutral loss filtering (NLF), and predictive database matching (DM) to decode the diterpenoid architectures (Gong et al., 2019; Liu et al., 2022; Ren et al., 2023; Wang et al., 2020; Zhou et al., 2024).

The specific structural analysis strategy for DAs is as follows: Step 1, Elemental Composition and RDB Profiling: A predictive DA database was constructed with defined elemental constraints (C: 0–60, H: 0–100, O: 0–20, N: 0–1; mass error <10 ppm). RDB values were calculated based on core skeletons and substituents: C19-Aconitine-type amino-alcohol DAs (C19-ADA) with saturated substituents (e.g., AC1–AC3 cores) exhibit an RDB of 5.5 for [M + H]^+^ ions. C20-Atisine-type, C20-Napelline-type, and C20-Hetisine-type DAs display RDB values of 5.5, 6.5, and 7.5, respectively. Unsaturated substituents modify the RDB: +1 (acetyl, C19-MDA), +4 (benzoyl, C19-MDA), or +5 (dual esters, C19-DDA). Additional RDB increments (+1) are introduced by dehydrogenated modifications (e.g., dehydro AC1–AC3 cores). Step 2, Neutral Loss Filtering and Substituent Identification: Key neutral losses (NL) were mapped to infer the presence of specific substituents: Hydroxyl (-H_2_O, 18 Da), methoxy (−CH_4_O, 32 Da), and acetyl ester (−C_2_H_4_O_2_, 60 Da). Combined with molecular formulas, NL patterns refine hypotheses about core structures and substitution patterns. Step 3, Diagnostic Ion Filtering for Core Differentiation: Characteristic fragment ions in CID-MS^2^ spectra differentiate DA subclasses. For example, base peak ions from C19-lipid-type DAs (C19-LDA) serve as markers for lipid-modified skeletons, while aromatic esters in C19-DDA/MDA generate distinct fragmentation patterns. Step 4, Database Matching and Novel Alkaloid Discovery: The DA database enables rapid annotation of known and predicted structures. Notably, C19-LDAs were redefined to include only long-chain fatty acid esters (>12 carbons), while short/medium-chain or aromatic esters were classified as C19-DDA/MDA. Tri-esterified DAs were categorized under C19-DDA, aligning structural taxonomy with bioactivity trends.

A total of 57 chemical components were identified across various processed products of aconite. These included 10 aconitine-type C19-UDA, 7 aconitine-type C19-MDA, 6 napelline-type C20, 6 aconitine-type C19-UDA, 5 sulfonated C20, 5 hetisine-type C20, 3 aconitine-type C19-MDA, 3 denudatine-type C20, 2 aconitine-type C19 glycoside-UDA, 2 franchetine-type C19, 2 zwitterionic sulfonated C20 (seco-napelline), 1 denudatine-type C20, 1 vakognavine-type C20, 1 aconitine-type C19 norditerpenoid, 1 aconitine-type C19-DDA, and 1 arcutine-type C20 (rare). The specific components are detailed in Table 1. This comprehensive analysis highlights the diverse chemical profile of aconite, offering valuable insights into how different processing methods influence the composition of DAs. This workflow not only accelerates DA characterization but also helps bridge gaps in understanding how processing methods (e.g., steaming, salt-baking) alter alkaloid profiles.

**TABLE 1:** Mass spectrometry identification data of chemical components in various processed products of aconitum.

Type of compound	Name of compound	Compound formula	AdductIon mode	m/z	MS^n^ information	t_R_/min	Mass error/ppm
hetisine-type C_20_	13β,15β,19α-trihydroxyhetisane	C_20_H_27_NO_3_	[M + H]^+^	330.2064	MS^2^ [330]: 312.1965, 294.1859 MS^3^ [330→294]: 276.1749	6.04	−4.6980
hetisine-type C_20_	hetisine	C_20_H_27_NO_3_	[M + H]^+^	330.2069	MS^2^ [330]: 312.1964 MS^3^ [300→312]: 294.1857, 276.1765	1.82	−3.6540
denudatine-type C_20_	aconicarnine A	C_20_H_29_NO_4_	[M + H]^+^	348.2175	MS^2^ [348]: 330.2068, 312.1961, 294.1855 MS^3^ [348→294]: 276.1738	4.87	−4.3270
hetisine-type C_20_	carmichaeline A	C_22_H_29_NO3	[M + H]^+^	356.2226	MS^2^ [356]: 296.2012 MS^3^ [356→296]: 278.1902	8.82	−3.9520
napelline-type C_20_	songoramine	C22H29NO_3_	[M + H]^+^	356.2226	MS^2^ [348]: 330.2432, 312.2325, 302.2117 MS^3^ [348→330]: 312.2327, 302.2119, 294.2195	5.48	−2.9870
napelline-type C_20_	songorine	C_22_H_31_NO_3_	[M + H]^+^	358.2382	MS^2^ [358]: 340.2274, 322.2171 MS^3^ [358→340]: 322.2170, 312.2326, 294.2227	1.44	−4.8720
napelline-type C_20_	12-epi-napelline	C_22_H_33_NO_3_	[M + H]^+^	360.2539	MS^2^ [360]: 342.2435 MS^3^ [360→342]: 324.2327, 312.2328, 298.2167, 280.2062	1.42	−4.4560
napelline-type C_20_	napelline	C_22_H_33_NO_3_	[M + H]^+^	360.2539	MS^2^ [360]: 342.2427 MS^3^ [360→342]: 324.2325, 314.2125, 306.2222	2.73	−5.6630
aconitine type C_19_-UDA	16β-hydroxycardiopetaline	C_21_H_33_NO_4_	[M + H]^+^	364.2488	MS^2^ [364]: 346.2379 MS^3^ [364→346]: 328.2279, 318.2073, 310.2173, 292.2072	1.72	−5.0830
hetisine-type C_20_	13β-acetoxy-15β,19α-dihydroxyhetisane	C_22_H_29_NO_4_	[M + H]^+^	372.2169	MS^2^ [372]: 354.2071, 312.1964, 294.1858 MS^3^ [372→312]: 294.1856, 284.2009, 266.1902	7.54	−4.0920
aconitine type C_19_-UDA	karakanine	C_22_H_33_NO_4_	[M + H]^+^	376.2488	MS^2^ [376]: 358.2380 MS^3^ [376→358]: 340.2274, 322.2163, 312.1961, 304.2063	3.32	−3.9820
denudatine-type C_20_	aconicarnine D	C_22_H_35_NO_4_	[M + H]^+^	378.2644	MS^2^ [378]: 360.2534 MS^3^ [378→360]: 342.2433, 332.2227, 324.2328, 306.2221	1.44	−5.7140
aconitine type C_19_-UDA	karakoline	C_22_H_35_NO_4_	[M + H]^+^	378.2644	MS^2^ [378]: 360.2180, 342.2070, 328.1914, 310.1807 MS^3^ [378→342]: 324.1964, 314.2118, 310.1806, 292.1696, 282.1857, 264.1768	11.57	−1.5340
aconitine type C_19_-UDA	aconicarchamine A	C_22_H_35_NO_4_	[M + H]^+^	378.2644	MS^2^ [376]: 358.2380 MS^3^ [376→358]: 340.2274, 322.2163, 312.1961, 304.2063	1.66	−1.4350
aconitine type C_19_-MDA	hokbusine B	C_22_H_33_NO_5_	[M + H]^+^	392.2437	MS^2^ [392]: 374.2328 MS^3^ [392→374]: 356.2220, 346.2009, 338.2119, 328.1924	1.82	−4.8120
denudatine-type C_20_	aconicarnine E	C_22_H_34_NO_5_	[M + H]^+^	392.2437	MS^2^ [392]: 374.2334, 356.2227, 344.2226, 338.2124 MS^3^ [392→374]: 356.2221, 344.2220, 338.2120, 326.2113	0.92	−3.6380
aconitine type C_19_-UDA	columbianine	C_22_H_35_NO_5_	[M + H]^+^	394.2593	MS^2^ [394]: 376.2488 MS^3^ [394→376]: 358.2379, 344.2223, 340.2276, 326.2118	1.77	−7.0120
denudatines C_20_	aconicarmine	C_22_H_35_NO_5_	[M + H]^+^	394.2593	MS^2^ [394]: 362.2331 MS^3^ [394→362]: 344.2221, 330.2069, 312.1963, 298.1804	6.31	−5.8210
aconitine type C_19_-MDA	N-ethylhokbusine B	C_24_H_37_NO_5_	[M + H]^+^	420.2750	MS^2^ [420]: 402.2642, 388.2487, 370.2382, 362.2330, 356.2226, 338.2120 MS^3^ [420→388]: 370.2379, 356.2224, 338.2118, 324.1962, 306.1859	4.59	−4.9710
C_20_ (sulfonated)	aconicatisulfonine A	C_22_H_31_NO_5S_	[M + H]^+^	422.1996	MS^2^ [422]: 404.2433 MS^3^ [422→404]: 386.2324, 372.2173, 354.2068, 340.1909, 322.1804, 304.1698	1.39	−4.5620
aconitine type C_19_-UDA	talatisamine	C_24_H_39_NO_5_	[M + H]^+^	422.2906	MS^2^ [422]: 404.2433 MS^3^ [422→404]: 386.2320, 372.2171, 326.2113, 312.1962	3.31	−4.3910
C_20_ (sulfonated)	aconicatisulfonine B	C_22_H_33_NO_5_S	[M + H]^+^	424.2158	MS^2^ [424]: 406.2593, 374.2331, 342.2068 MS^3^ [424→406]: 388.2484, 37	3.79	−3.5210
aconitine-type C_19_-UDA	delstaphisine 8,14-deacetate	C_23_H_37_NO_6_	[M + H]^+^	424.2699	MS^2^ [424]: 406.2595, 388.2488, 356.2226 MS^3^ [424→406]: 3 356.2222, 338.2115, 324.1959, 306.1852	1.52	−5.5310
aconitine-type C_19_-UDA	senbusine A	C_23_H_37_NO_6_	[M + H]^+^	424.2699	MS^2^ [424]: 406.2596, 392.2437, 374.2334 MS^3^ [424→392]: 374.2327, 360.2169, 342.2066, 310.1802	4.29	−3.6348
aconitine-type C_19_-UDA	senbusine B	C_23_H_37_NO_6_	[M + H]^+^	424.2699	MS^2^ [424]: 406.2594 MS^3^ [424→406]: 356.2224, 342.2068, 324.1959, 310.1806, 292.1707	6.78	−3.6720
C_20_ (sulfonated)	aconicarmisulfonine A	C_22_H_29_NO_6_S	[M + H]^+^	436.1794	MS^2^ [436]: 418.2227 MS^3^ [436→418]: 400.2127, 386.1967, 368.1864, 358.2017, 354.1705	5.11	−3.4930
franchetine-type C_19_	guiwuline	C_24_H_37_NO_6_	[M + H]^+^	436.2699	MS^2^ [436]: 418.2581, 404.2432, 386.2328, 376.2123 MS^3^ [436→404]: 386.2327, 376.2120, 326.2122, 322.1795, 294.1858	6.96	−2.4377
franchetine-type C_19_	carmichasine C	C_24_H_37_NO_6_	[M + H]^+^	436.2699	MS^2^ [436]: 418.2589 MS^3^ [436→418]: 400.2485, 390.2279, 386.2322, 368.2223, 340.2275, 308.2005, 290.1932	5.11	−3.5410
C_20_ (sulfonated)	aconicarmisulfonine B	C_22_H_31_NO_6_S	[M + H]^+^	438.1950	MS^2^ [438]: 420.2744, 406.2590, 388.2483, 356.2222 MS^3^ [438→388]: 370.2381, 356.2225, 338.2114, 324.1963, 306.1856	3.81	−5.1800
C_20_ (sulfonated)	aconicarmisulfonine C	C_22_H_31_NO_6_S	[M + H]^+^	438.1950	MS^2^ [438]: 420.2744, 406.2588, 390.2638 MS^3^ [438→420]: 402.2641, 388.2482, 370.2401, 356.2216, 338.2106	7.38	−4.7600
aconitine-type C_19_-UDA	neoline	C_24_H_39_NO_6_	[M + H]^+^	438.2856	MS^2^ [438]: 420.2759 MS^3^ [438→420]: 402.2643, 388.2487, 370.2383, 356.2227, 338.2121, 324.1964, 306.1859, 288.1752	4.51	−4.7560
aconitine-type C_19_-UDA	subcusine	C24H39NO6	[M + H]^+^	438.2856	MS^2^ [438]: 420.2388 MS^3^ [438→420]: 402.2282, 388.2125, 374.2333, 342.2070, 310.1807	5.59	−2.0120
aconitine-type C_19_-UDA	foresticine	C_24_H39NO6	[M + H]^+^	438.2856	MS^2^ [438]: 420.2747, 406.3593, 388.2486, 356.2224 MS^3^ [438→406]: 388.2482, 370.2378, 356.2222, 338.2116, 324.1958, 306.1855, 288.1740	5.13	−3.6360
hetisine-type C20	(−)-(13R,19S)-11α,19-dihydroxy-N-methyl-13-(S-2-methylbutyryloxy)-2α-hydroxyhetisanium	C_26_H_38_NO_5+_	[M + H]^+^	444.2750	MS^2^ [444]: 426.2280 MS^3^ [444→426]: 408.2190, 342.1705, 324.1597, 306.1488, 296.1642, 288.1385, 278.1535, 260.1435	9.21	−3.8930
aconitine-type C_19_-UDA	fuziline	C_24_H_39_NO_7_	[M + H]^+^	454.2805	MS^2^ [454]: 436.2699 MS^3^ [454→436]: 418.2586, 404.2429, 386.2324, 354.2065, 322.1804, 304.1694	1.99	−2.8700
aconitine-type C_19_-UDA	delcosine	C_24_H_39_NO_7_	[M + H]^+^	454.2805	MS^2^ [454]: 436.2697, 422.2540, 418.2591, 386.2328, 372.2172 MS^3^ [454→418]: 354.2066, 368.2219, 336.1962, 322.1800	2.61	−7.0910
zwitterionic sulfonated C_20_	aconapelsulfonine A	C_22_H_33_NO_8_S	[M + H]^+^	472.1992	MS^2^ [472]: 412.2483 MS^3^ [472→412]: 328.1907, 310.1802, 292.1695, 274.1594	11.20	−4.0290
zwitterionic sulfonated C_20_	aconapelsulfonine B	C_22_H_33_NO_8_S	[M + H]^+^	472.1992	MS^2^ [472]: 454.2606, 428.2437, 412.2486, 368.2224, 354.2069, 296.2013 MS^3^ [472→354]: 336.1961, 312.1968, 294.1857, 276.1749	12.40	−3.5270
aconitine-type C_19_-UDA	N-deethylaconine	C_23_H_37_NO_9_	[M + H]^+^	472.2547	MS^2^ [472]: 370.2014 MS^3^ [472→370]: 352.1911, 342.2067, 310.1804, 292.1698, 282.1857, 264.1749	12.83	−4.5590
aconitine-type C_19_-UDA	beiwutinine	C_24_H_39_NO_10_	[M + H]^+^	502.2652	MS^2^ [502]: 458.2542 MS^3^ [502→458]: 370.2015, 312.1962	9.76	−3.6780
vakognavine-type C_20_	carmichaeline C	C_29_H_41_NO_7_	[M + H]^+^	516.2961	MS^2^ [516]: 472.2694 MS^3^ [516→472]: 370.2013, 312.1961	11.00	−3.2510
aconitane-type C_19_ glycoside-UDA	aconicarmichoside D	C_29_H_47_NO_10_	[M + H]^+^	520.3512	MS^2^ [520]: 358.2375, 340.2272 MS^3^ [520→340]: 322.2173, 312.2327	2.62	−4.7980
aconitine-type C_19_-MDA	carmichaeline L	C_30_H_41_NO_7_	[M + H]^+^	528.2961	MS^2^ [528]: 510.2701, 496.2539, 478.2435, 464.2284, 446.2178 MS^3^ [528→478]: 414.1910, 404.2062, 396.1796, 386.1967, 368.1860, 354.1705	3.81	−3.8950
aconitine-type C_19_-MDA	14-O-cinnamoylneoline	C_33_H_45_NO_7_	[M + H]^+^	568.3274	MS^2^ [568]: 550.3011, 536.2856, 518.2756, 504.2603, 486.2491 MS^3^ [568→518]: 454.2224, 436.2119, 418.2231, 386.1973, 354.1697, 336.1592	10.20	−4.6010
aconitine-type C_19_-MDA	8-O-cinnamoylneoline	C_33_H_45_NO_7_	[M + H]^+^	568.3274	MS^2^ [568]: 420.2752, 402.2648 MS^3^ [568→420]: 402.2645, 370.2383, 338.2119, 306.1855	16.40	−3.6890
aconitane-type C_19_ glycoside-UDA	aconicarmichoside A	C_29_H_47_NO_10_	[M + H]^+^	570.3278	MS^2^ [570]: 552.2963, 534.2859 MS^3^ [570→552]: 470.2325, 452.2219, 404.2433, 386.2328, 372.2176, 354.2068, 336.1961, 322.1808, 318.1831	13.87	−3.7021
aconitine-type C_19_-MDA	Aconitane-6,8,13,14,15,16-hexol, 1-methoxy-4-(methoxymethyl)-20-methyl-, 8-acetate 14-benzoate, (1α,6α,14α,15α,16β)	C_31_H_41_NO_10_	[M + H]^+^	588.2809	MS^2^ [588]: 556.2540 MS^3^ [588→556]: 524.2283, 506.2185, 470.2175, 452.2059, 438.1911, 352.1552	10.1	−4.6010
aconitine type C_19_-MDA	benzoyldeoxyaconine	C_32_H_45_NO_9_	[M + H]^+^	588.3173	MS^2^ [588]: 556.2545 MS^3^ [588→556]: 524.2285, 506.2181, 492.2011, 470.2174, 452.2070, 438.1913, 422.1961, 402.1913, 384.1823, 370.1641, 352.1566, 324.1598, 310.1443, 292.1343	11	−2.2500
aconitine-typeC_19_ norditerpenoid	3-deoxyhokbusine A	C_32_H_45_NO_9_	[M + H]^+^	588.3173	MS^2^ [588]: 570.2561, 556.2408, 538.2366 MS^3^ [588→538]: 520.2292, 506.2109, 474.1871, 416.2409, 384.2082, 366.1782, 352.1614, 334.1447, 320.1640, 302.1164	13.3	−3.3110
aconitine-type C_19_-MDA	14-O-veratroylneoline	C_33_H_47_NO_9_	[M + H]^+^	602.3329	MS^2^ [602]: 570.2699, 542.2745, 510.2484 MS^3^ [602→542]: 524.2648, 510.2488, 482.2540, 450.2275, 420.2386, 402.2292, 388.2131, 370.2022, 356.1858, 338.1754, 324.1598, 306.1485	13.49	−4.2610
aconitine-type C_19_-MDA	neojiangyouaconitine	C_33_H_47_NO_9_	[M + H]^+^	602.3329	MS^2^ [602]: 570.2695, 556.2913, 524.2651 MS^3^ [602→556]: 464.2443, 432.2180, 402.2286, 370.2016, 338.1759	15.09	−4.4900
aconitine-type C_19_-MDA	(−)-(A-b)-14α-benzoyloxy-N-ethyl-13β,15α-dihydroxy-1α,6α,8β,16β,18-pentamethoxyaconitane	C_33_H_47_NO_9_	[M + H]^+^	602.3329	MS^2^ [602]: 584.2849, 570.2698, 552.2581, 542.2747, 524.2646 MS^3^ [602→542]: 482.2535, 464.2441, 450.2271, 432.2193, 420.2383, 402.2274	15.59	−4.2720
aconitine-type C_19_-DDA	carmichaeline M	C_34_H_47_NO_9_	[M + H]^+^	614.3329	MS^2^ [614]: 554.2750 MS^3^ [614→554]: 522.2483, 494.2535, 372.2172, 344.1878, 312.1596, 280.1336	14	−3.6950
napelline-type C_20_	aconicarmichinium B	C_22_H_30_NO_3_	[M + H]^+^	356.2226	MS^2^ [356]: 338.2122, 328.2277, 312.1966, 296.1651 MS^3^ [356→296]: 278.1542, 268.1700, 250.1603	0.91	−3.5400
arcutine-type C_20_ (rare)	aconicarmicharcutinium A	C_22_H_32_NO_3_	[M + H]^+^	358.2382	MS^2^ [358]: 340.2274, 322.2171 MS^3^ [358→340]: 322.2170, 312.2326, 294.2227	1.38	−4.8000
napelline-type C_20_	aconicarmichinium A	C_22_H_32_NO_3_	[M + H]^+^	358.2382	MS^2^ [358]: 340.2274 MS^3^ [358→340]: 322.2169, 312.2324, 304.2070	2.51	−4.1490

### 3.3 Determination of 12 active ingredients in different Fuzi products: exploring the relationship between processing method and retention of efficacy components

**FIGURE 5:**
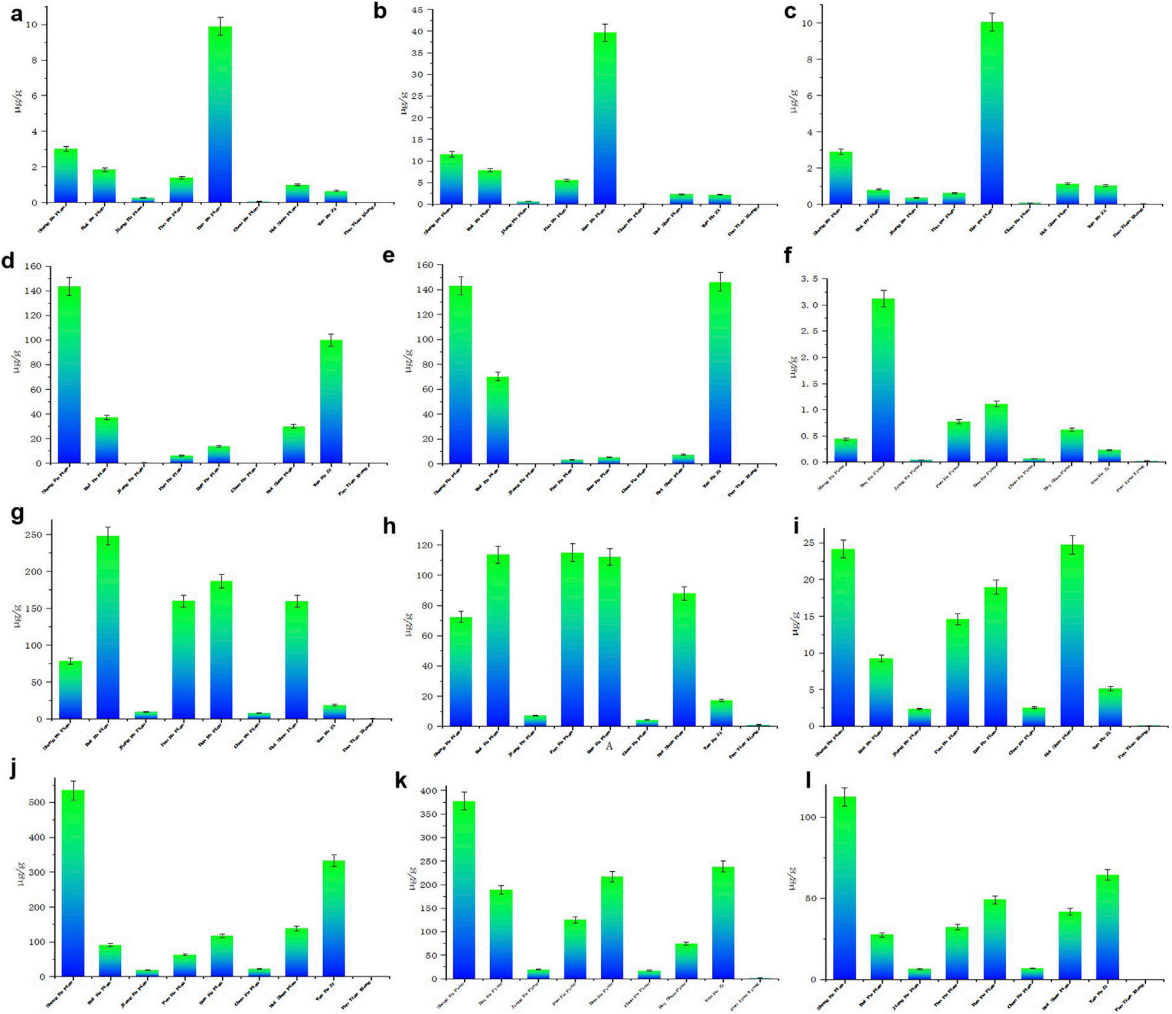
Determination results of 12 aconitum alkaloids in various processed products of Fuzi (μg/g) **(a)** Aconine, **(b)** Mesaconine, **(c)** Hypaconine, **(d)** Hypaconitine, **(e)** Mesaconitine, **(f)** Lappaconitine, **(g)** Benzoylaconine, **(h)** Benzoylmesaconine, **(i)** Benzoylhypaconine, **(j)** Karacoline, **(k)** Fuziline, **(l)** Songorine.

Accurately weighed samples of Aconitum carmichaelii prepared using nine different processing methods were used to prepare test solutions according to the respective methods. The contents of 12 active components were then quantified, and the results are presented in Figure 5. The findings revealed significant variations in the levels of these 12 compounds across different processed varieties of *Radix Aconiti Lateralis Preparata*. The total content of the 12 components, ranked from highest to lowest, is as follows: Sheng Fu Pian, Yan Fu Zi, Bai Fu Pian, Dan Fu Pian, Hei Shun Pian, Pao Fu Pian, Jiang Fu Pian, Chao Fu Pian, and Pao Tian Xiong. Alkaloid contents were imported into SIMCA 13.03 software for principal component analysis (PCA). The results, shown in Figures 4a,b indicate significant differences in alkaloid profiles among the processed products. Notably, Yan Fu Zi and Sheng Fu Pian exhibited minimal differences, suggesting that different processing techniques have a relatively consistent impact on alkaloid content.

Apart from Bai Fu Pian and Hei Shun Pian, distinct differences in alkaloid content were observed due to processing techniques such as frying, baking, and calcination, as illustrated in Figure 4c. Variable importance in projection (VIP) was used as an indicator to identify alkaloids that significantly influenced the content of active components in Aconitum carmichaelii across different processing methods. Alkaloids with VIP >1, including mesaconine, lappaconitine, benzoylmesaconine, benzoylhypaconine, and fuziline, were identified as key markers for evaluating the processing methods of *Radix Aconiti Lateralis Preparata*.

**FIGURE 6:**
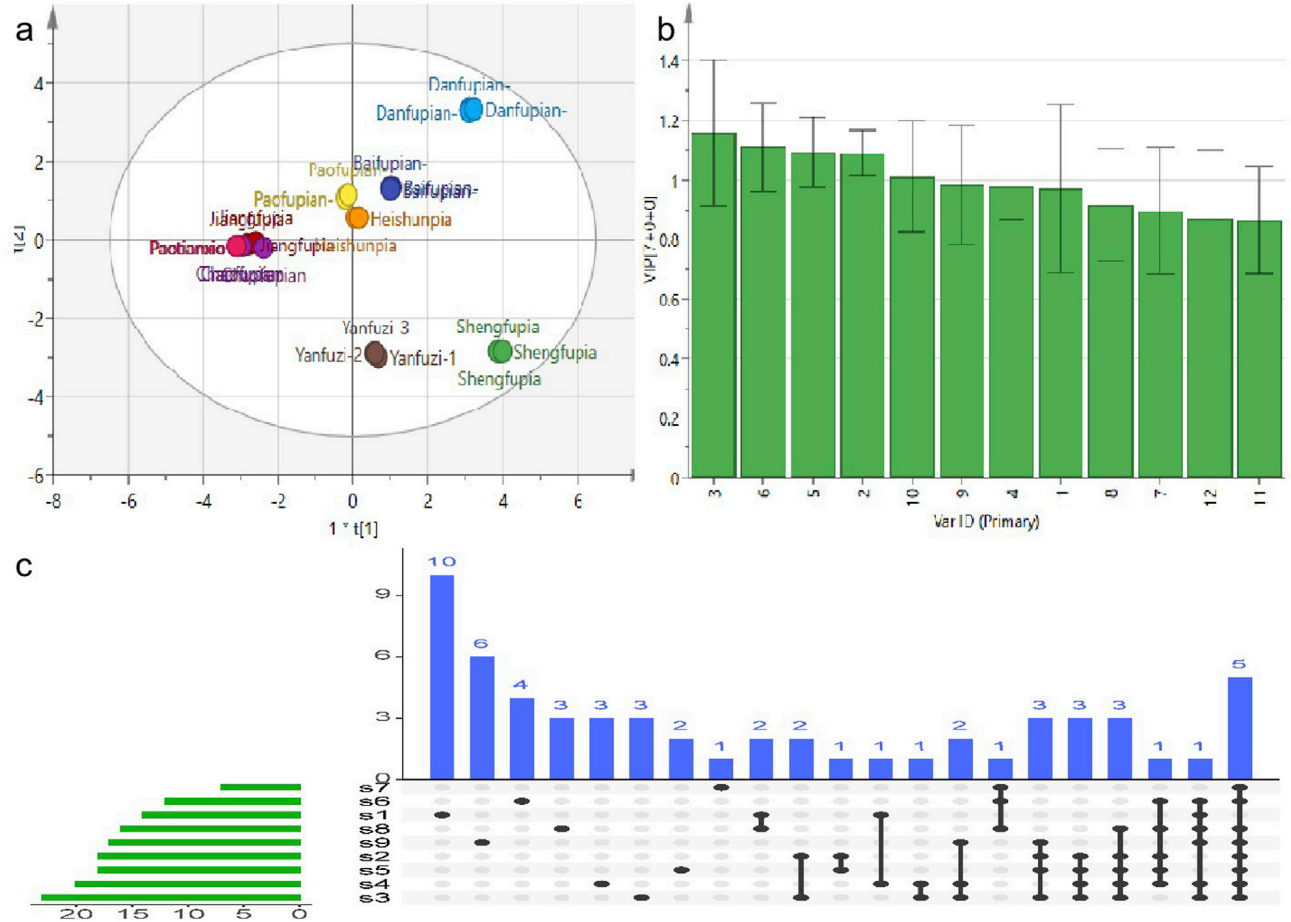
OPLS-DA and component association of different processed products of Fuzi **(a)** OPLS-DA score plot of the association between 12 active components in various processed products, **(b)** OPLS-DA VIP plot of various processed products, **(c)** UpSet diagram of different processed products. S1-S9 represent Sheng Fu Pian, Bai Fu Pian, Jiang Fu Pian, Pao Fu Pian, Dan Fu Pian, Chao Fu Pian, Hei Shun Pian, Yan Fu Zi, and Pao Tian Xiong, respectively.

The OPLS-DA score plot (Figure 6a) provides a visual representation of the relationships between the 12 active components in the different processed products of Aconitum carmichaelii. This plot highlights distinct clustering patterns, reflecting the unique chemical profiles of each processed form. The separation of these clusters indicates that the processing methods significantly alter the chemical composition of the herbs. The OPLS-DA model (Figure 6a) demonstrated strong predictive capability, with fit parameters R^2^ = 0.683 and Q^2^ = 0.762, indicating excellent model stability and predictive power. Permutation testing (200 iterations; Supplementary Figure S2) further confirmed the model’s validity: all permuted R^2^ and Q^2^ values (left distribution) were lower than the original model values (right distribution). This rigorous permutation validation conclusively demonstrates the absence of overfitting. The OPLS-DA VIP plot (Figure 6b) identifies the most influential variables contributing to the differentiation of the processed products. Variables with VIP values greater than 1 are considered significant, highlighting their importance in distinguishing between the various forms of Aconitum carmichaelii. Key compounds such as mesaconine, lappaconitine, benzoylmesaconine, benzoylhypaconine, and fuziline show high VIP values, indicating their critical role in the chemical variability of the processed products. The UpSet diagram (Figure 6c) provides a detailed overview of the common and unique active ingredients across the different processed products. This diagram helps identify shared and distinct components, offering insights into the chemical diversity and potential therapeutic effects of each processed form. The UpSet diagram is particularly valuable for understanding the complex interactions and overlaps among the active components in the various preparations.

Collectively, these analyses offer a comprehensive framework for evaluating the impact of different processing methods on the chemical composition of Aconitum carmichaelii, emphasizing the importance of precise analytical techniques in ensuring the quality and efficacy of traditional herbal medicines.

### 3.4 Study on anti-tumor activity of different Fuzi products: processing method determines efficacy and safety

**FIGURE 7:**
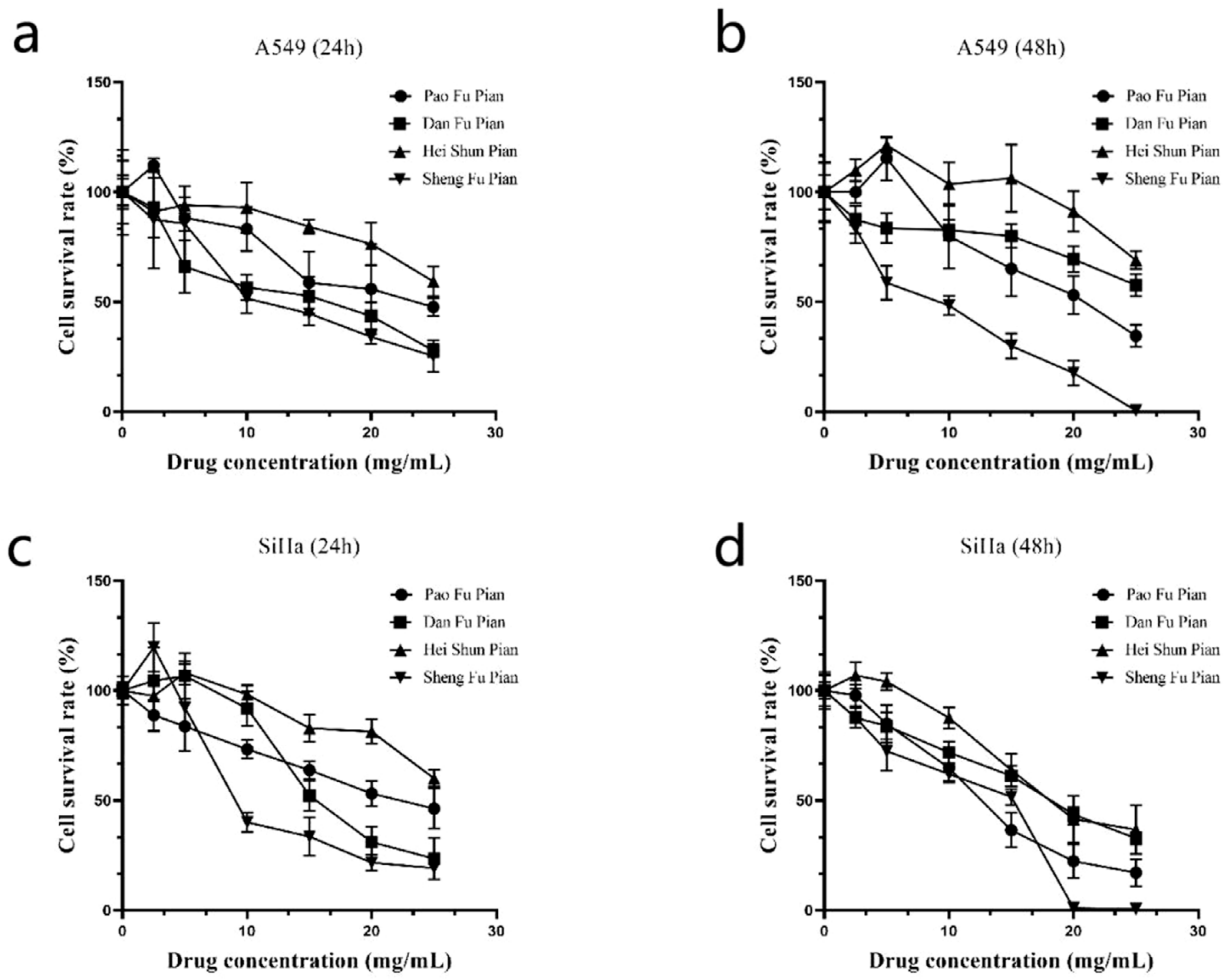
Inhibitory effects of four processed products of Fuzi on A549 and SiHa cells (n = 3 independent biological replicates).

To assess the therapeutic efficacy of different processed Aconitum carmichaelii products, the *in vitro* anti-cancer activity of four commonly used forms (Sheng Fu Pian, Dan Fu Pian, Pao Fu Pian, and Hei Shun Pian) was evaluated using the CCK-8 assay on human lung adenocarcinoma (A549) and cervical carcinoma (SiHa) cells. All processed products significantly inhibited cell proliferation in both cancer cell lines (Figure 7). Notably, Sheng Fu Pian exhibited the strongest cytotoxicity at both 24 and 48 h, while Hei Shun Pian showed the weakest activity. Dan Fu Pian and Pao Fu Pian demonstrated intermediate inhibitory effects. These results highlight a strong correlation between anti-cancer efficacy and chemical compositional changes resulting from different processing methods. The observed variations in anti-cancer activity underscore the critical role of processing techniques in modulating the bioactive components of Aconitum carmichaelii. This mechanistic insight is crucial for optimizing clinical applications and ensuring consistent efficacy in traditional herbal formulations.

## 4 Discussion

Over the centuries, various processing methods for Fuzi have been developed, each yielding distinct properties and chemical compositions due to differences in processing techniques. The present study aimed to investigate the impact of different processing methods on the properties, chemical composition, and active ingredients of Fuzi, including Sheng Fu Pian, Yan Fu Zi, Dan Fu Pian, Bai Fu Pian, Jiang Fu Pian, Pao Fu Pian, Chao Fu Pian, Hei Shun Pian, and Pao Tian Xiong.

SEM imaging (Figure 2) revealed the complete disintegration of starch granules in steamed products (Hei Shun Pian), forming a hydrogel network that physically encapsulates toxic diester alkaloids, such as aconitine. This encapsulation delays gastrointestinal release, thereby reducing acute toxicity while preserving cardiotonic monoester alkaloids—striking a balance between detoxification and efficacy.

SEM and XRD morphometric analysis uncovered processing-induced alterations in starch ultrastructure in Fuzi preparations. The results demonstrated that starch granules in Fuzi undergo substantial modifications depending on the processing method. Specifically, the natural crystalline structure of Aconitum starch was completely disintegrated during processing, and the morphology of the starch granules varied with the processing technique. SEM imaging (Figure 2) indicated that steamed products (Hei Shun Pian) retained higher levels of diester-DAs (e.g., aconitine) compared to dry-roasted varieties (Chao Fu Pian). This discrepancy can be attributed to the formation of a hydrogel network in steamed Fuzi, which physically encapsulates toxic alkaloids and retards their dissolution. This barrier effect delays the release of alkaloids in the gastrointestinal tract, reducing acute toxicity while prolonging therapeutic effects. The degree of gelatinization plays a pivotal role in balancing toxicity and efficacy: starch forms a keratinized matrix that minimizes alkaloid leaching while facilitating the hydrolysis of toxic diester alkaloids into monoester derivatives, achieving simultaneous detoxification and preservation of cardiotonic compounds. Concomitant XRD analysis (Figure 3) showed that raw Fuzi contains abundant needle-shaped calcium oxalate raphides, which likely contribute to local mucosal irritation, such as oral numbness. Processing disrupts these crystalline structures, reducing physical irritancy while increasing the exposure of alkaloids to hydrolytic conditions—further mitigating systemic toxicity. Collectively, our findings demonstrate that starch gelatinization governs detoxification by physically encapsulating toxic alkaloids and reducing leaching, while crystal destruction enhances efficacy preservation by promoting the hydrolysis of alkaloids. Additionally, the keratinized starch structure provides resistance to insect infestation, enabling long-term storage and ensuring consistent clinical efficacy. These results highlight the need for further standardization of Fuzi processing methods to ensure both safety and consistency. Potential heavy metal contamination from processing adjuvants (e.g., calcium chloride in Danba processing) was not quantified. Given that Yan Fu Zi and similar salt-processed variants retained high levels of sodium and chloride ions (Figure 3), future QC protocols should include heavy metal screening to ensure safety, particularly for long-term use.

To elucidate the dynamic relationship between toxic compound degradation and bioactive constituent retention, this study employed metabolomics to investigate the structural remodeling of DAs in processed Aconitum products. This approach revealed the material basis underlying reduced toxicity and enhanced efficacy. Building on this metabolic profiling, 12 bioactive compounds were quantified across different processing methods to evaluate their critical influence on therapeutic outcomes. LC-MS metabolomics (Figure 4) identified 57 DAs, and subsequent quantification highlighted method-dependent transformations: Steaming and boiling preferentially hydrolyzed C19-diester alkaloids, evidenced by a 92% reduction in aconitine in Pao Tian Xiong compared to raw aconite. Salt-baking promoted sulfonation of C20-alkaloids, converting neurotoxic diesters into anti-inflammatory monoesters, as shown by an 8.7-fold increase in aconitarmisulfonine B in Yan Fu Zi. CCK-8 assays (Figure 7) validated these transformations: elevated cytotoxicity in Sheng Fu Pian correlated with remaining diester alkaloids, while the safety profile of Hei Shun Pian, derived from sulfonated alkaloids comprising 82% of its alkaloid fraction, demonstrated a reduced toxicity profile. Systematic evaluation of processed Aconitum carmichaelii products revealed significant differences in clinical efficacy and safety, with processing methods identified as the key determinant of therapeutic indices. This finding highlights the importance of selecting optimized processing protocols to balance pharmacological activity and toxicity reduction, informing evidence-based clinical decisions for this widely used traditional herbal remedy.

This “structure-chemistry-efficacy nexus” provides regulatory bodies with a practical framework for refining processing protocols, moving beyond empirical approaches toward mechanism-based optimization of herbal medicine standardization. Our XRD analyses (Figure 3), showing peak elimination corresponding to starch crystallites, align with Zhou et al. (2015), who reported similar crystalline disruption in steamed Fuzi. However, our integrated SEM/Metabolomics approach offers a significant advancement: sulfonation acts as a co-driver of detoxification, contrasting with Liu et al. (2017), who attributed this process solely to ester hydrolysis. This divergence from previous studies contrasts with Lei et al. (2021), whose LC-MS methodology failed to detect sulfonated derivatives due to sensitivity limitations. Moreover, while Wang et al. (2020) reported inconsistent anti-cancer effects across processed products, our CCK-8 assays systematically establish correlations between cytotoxicity and alkaloid profiles. This resolves longstanding controversies—particularly regarding why Dan Fu Pian exhibits superior analgesic efficacy compared to Chao Fu Pian, a difference attributed to its higher retention of monoester alkaloids. While the CCK-8 assays demonstrated significant anti-cancer effects across processed products, this study was limited to *in vitro* models (A549 and SiHa cells). Future work should validate efficacy *in vivo*, particularly for indications aligned with clinical applications of specific Fuzi (e.g., Dan Fu Pian for analgesia or Pao Fu Pian for Yang deficiency). Additionally, the 48-h exposure may not capture chronic toxicity, warranting longer-term studies.

This study systematically elucidated the effects of nine distinct processing methods on the microstructural characteristics, chemical composition, bioactive constituent profiles, and pharmacodynamic properties of Fuzi. By integrating SEM analysis, chemical marker quantification (e.g., diester-DAs), and bioactivity evaluation, this study revealed the dynamic equilibrium between toxin degradation and therapeutic component preservation during processing. This multidimensional approach advances the scientific foundation of herbal processing theory, offering comprehensive strategies for optimizing Fuzi processing protocols, elucidating pharmacologically active constituents, and providing critical references for clinical application with balanced efficacy and safety profiles. This understanding will facilitate the standardization and optimization of Fuzi processing methods, ultimately enhancing its safe and effective use in clinical practice. It is also noteworthy that certain processing methods (Hei shun pian) employ Danba to reduce the toxicity of Fuzi. While this industrial approach mitigates acute toxicity, it introduces residual heavy metals—particularly lead, mercury, and aluminum end—which may cause gastrointestinal disturbances and chronic toxicity. Moreover, excessive Danba use risks diminishing therapeutic efficacy. Future protocols must standardize rinsing procedures, establish elemental residue thresholds, and promote Danba-free alternatives to balance safety and pharmacological activity.

## Data Availability

The original contributions presented in the study are included in the article/Supplementary Material, further inquiries can be directed to the corresponding authors.
