# Supplementary material for: Deciphering structure-composition-efficacy transformation in processed Fuzi: an integrated SEM, XRD, metabolomics, and pharmacodynamics approach
Source: Front Chem. 2025 Sep 11;13:1630159. doi: 10.3389/fchem.2025.1630159 (PMC12460371; doi:10.3389/fchem.2025.1630159)
Supplement: Supplementary file 1 [file DataSheet1.docx]

**Supplementary Materials**

Dear Editors,

Please find the Supplementary Materials for our manuscript entitled "[Deciphering Microstructural-Chemical-Efficacy Disparities in Processed Aconite via Integrated SEM and Multi-omics Profiling]" . These materials include:

Supplementary Figures/Tables: Extended data supporting the main findings (e.g., additional spectra, chromatograms, or statistical analyses).

Method Details: Extended protocols for reproducibility (e.g., synthesis procedures or computational methods).

All materials are referenced in the main text and comply with Molecules' guidelines. They are intended to enhance transparency and support the conclusions without duplicating the main content.

Sincerely Yours.

Dr. Qinhua Chen

Date: March 30, 2025

**Supplementary Figures**

**
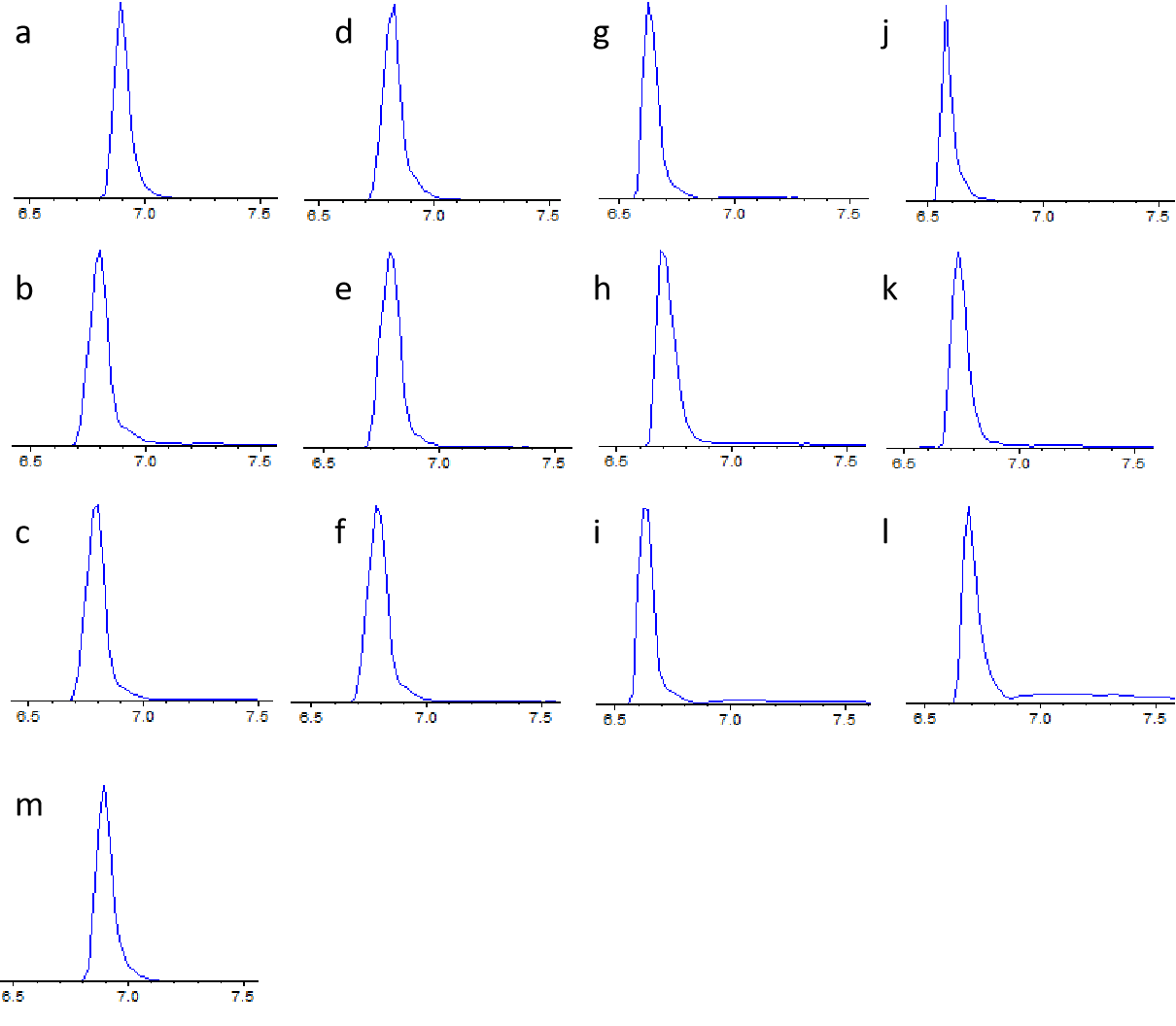
**

**Fig S1 The specificity of 12 reference standards and 1 internal standard**

（a：Hypaconitine；b：Lappaconitine；c：Benzoylmesaconine；d：Mesaconitine；e：Benzoylaconine；f：Benzoylhypaconine；g：Aconine；h：Hypaconine；i：Karacoline；j：Mesaconine；k：Fuziline；l：Songorine；m：Yohimbine.）

**Supplementary Table**

**Table S1 Information on 13 standard**

| **Name** | **Source** | **CAS** | **Fineness** |
| --- | --- | --- | --- |
| Aconine | MUST-22070306 | [509-20-6](https://www.chembk.com/cn/chem/509-20-6) | Chengdu Minster Biotechnology Co., Ltd., China |
| Mesaconine | MUST-23061504 | 6792-09-2 | Chengdu Minster Biotechnology Co., Ltd., China |
| Hypaconine | MUST-22082618 | [63238-68-6](https://www.chembk.com/cn/chem/63238-68-6) | Chengdu Minster Biotechnology Co., Ltd., China |
| Hypaconitine | MUST-231032112 | [6900-87-4](https://www.chembk.com/cn/chem/6900-87-4) | Chengdu Minster Biotechnology Co., Ltd., China |
| Mesaconitine | MUST-22120319 | [2752-64-9](https://www.chembk.com/cn/chem/2752-64-9) | Chengdu Minster Biotechnology Co., Ltd., China |
| Lappaconitine | MUST-23050119 | [32854-75-4](https://www.chembk.com/cn/chem/32854-75-4) | Chengdu Minster Biotechnology Co., Ltd., China |
| Benzoylaconine | MUST-22102110 | [466-24-0](https://www.chembk.com/cn/chem/466-24-0) | Chengdu Minster Biotechnology Co., Ltd., China |
| Benzoylmesaconine | MUST-22102410 | [63238-67-5](https://www.chembk.com/cn/chem/63238-67-5) | Chengdu Minster Biotechnology Co., Ltd., China |
| Benzoylhypaconine | DB-22121912 | 63238-66-4 | China lemeitian pharmaceutical ｜ Dexter biology |
| Karacoline | MUST-21052821 | [39089-30-0](https://www.chembk.com/cn/chem/39089-30-0) | Chengdu Minster Biotechnology Co., Ltd., China |
| Fuziline | MUST-22072617 | [80665-72-1](https://www.chembk.com/cn/chem/80665-72-1) | Chengdu Minster Biotechnology Co., Ltd., China |
| Songorine | MUST-23083613 | [509-24-0](https://www.chembk.com/cn/chem/509-24-0) | Chengdu Minster Biotechnology Co., Ltd., China |
| Yohimbine | MUST-21072710 | 146-48-5 | Chengdu Minster Biotechnology Co., Ltd., China |

**Table S2 *Aconiti Lateralis Radix Praeparata* A Comprehensive Compilation of Nine Distinct Preparation Methodologies**

|  | **Preparation method** | **Characteristic sheet type** | **Prescription norms** |
| --- | --- | --- | --- |
| **Sheng Fu Pian** | Take the mud and aconite, wash it, slice it and dry it | The epidermis is yellowish-brown or dark brown, and the cross section is white or light gray-yellow. It tastes pungent and numb; it is light and brittle. | Chinese Pharmacopoeia 2020 edition |
| **Bai Fu Pian** | Select the selected aconite and scald it with sand until it bulges and slightly changes color. | No skin, yellowish white, translucent, bulging yellow brown on the surface, brittle. | Chinese Pharmacopoeia 2020 edition |
| **Jiang Fu Pian** | Take the salted aconite root, wash it with clean water for 3 days, changing the water 2 to 3 times daily until all the salt is removed. Scrape off the outer skin with a knife and rinse thoroughly with clean water. Cut into 0.3~0.5cm thick slices horizontally, then soak in rice washing water for 3 days. Remove the ginger slices and mix them evenly. Place the large slices in the center of the wooden steamer and the smaller slices around the edges. Steam for 6 to 8 hours until the surface shows oiliness. Pour into bamboo sieves, spread flat, and fan to create a "crust" on the surface. Dry over low heat. | The section is light yellow-brown, translucent and keratinous. It is firm in texture. It has a slight smell and a bland taste. It is an irregular longitudinal slice about 0.3~0.5cm thick. | Standard for Processing of Traditional Chinese Medicine in Jiangxi Province, 2008 |
| **Pao Fu Pian** | Select the selected aconite and scald it with sand until it bulges and slightly changes color. | No skin, yellowish white, translucent, bulging yellow brown on the surface, brittle. | Chinese Pharmacopoeia 2020 edition |
| **Dan Fu Pian** | Select salted aconite, wash away the salt, and cook it with licorice and black beans until the taste is not bitter. Then cut it into thin slices and dry it in the sun. | The epidermis is dark brown, with a cross section of brown and translucent; it is hard, with a cuticle-like fracture, and no tongue sensation when tasted. | Anhui Province Standard for Processing Chinese Herbal Decoctions, 2005 edition |
| **Chao Fu Pian** | Select raw aconite and stir-fry it until smooth. Then put raw aconite slices into the sand until the skin is yellowish-brown and the cross section is yellow. | The epidermis is yellowish-brown, the cross section is light yellow or yellowish-brown, the texture is loose and crisp; the taste is slightly bitter, with a slight tingling sensation. | Sichuan Chinese Medicine Processing Standard, 2015 edition |
| **Hei Shun Pian** | Select the selected aconite and boil until it is transparent; steam until the surface appears glossy and dry. | The epidermis is dark brown, the cross section is dark yellow, translucent, hard and brittle, and the section is keratinous. | Chinese Pharmacopoeia 2020 edition |
| **Yan Fu Zi** | After soaking the mud aconite in bile water overnight, add salt and continue to soak it. Take it out every day and dry it in the sun, and gradually extend the drying time until a large number of crystalline salt particles (salt frost) appear on the surface of the aconite and the body becomes hard. Cut it into thick slices and dry it. | The surface is covered with salt frost, grayish black, with bud scars at the top and tumor-like protrusions or scars of lateral roots around it; small voids filled with salt frost and polygonal ring patterns of the forming layer can be seen; it tastes salty and numb, and stings the tongue. | Guangxi Zhuang Autonomous Region Standard for Processing Traditional Chinese Medicine, 2007 edition |

**Table S3 The MS parameters of the 12 components and internal standard (IS)**

| **Name of compound** | **parent ion** | **daughter ion** | **DP（eV)** | **CE（eV)** | **Retention time (min)** |
| --- | --- | --- | --- | --- | --- |
| **Aconine** | 500.3 | 450.1 | 100 | 45 | 6.65 |
| **Mesaconine** | 486.1 | 436.1 | 85 | 35 | 6.61 |
| **Hypaconine** | 470.3 | 438.2 | 85 | 25 | 6.74 |
| **Hypaconitine** | 616.3 | 556.2 | 95 | 45 | 6.91 |
| **Mesaconitine** | 632.4 | 572.2 | 100 | 45 | 6.82 |
| **Lappaconitine** | 585.5 | 535.3 | 70 | 30 | 6.81 |
| **Benzoylaconine** | 604.5 | 554.4 | 115 | 50 | 6.78 |
| **Benzoylmesaconine** | 590.3 | 540.2 | 65 | 45 | 6.82 |
| **Benzoylhypaconine** | 574.4 | 542.3 | 95 | 45 | 6.79 |
| **Karacoline** | 378.2 | 360.2 | 80 | 20 | 6.63 |
| **Fuziline** | 454.2 | 436.3 | 85 | 25 | 6.75 |
| **Songorine** | 358.3 | 340.3 | 75 | 25 | 6.69 |
| **Yohimbine (IS)** | 355.2 | 212.3 | 75 | 35 | 6.92 |

**Table S4. Regression equations and linear ranges for the 12 components**

| **Name of compound** | **Regression equations** | **r** | **Range (ng/mL)** | **Detectability** | **Quantitative limit** |
| --- | --- | --- | --- | --- | --- |
| Aconine | Y=1.05002e5x+4864.22812 | 0.99524 | 0.25-516 | 0.01 | 0.03 |
| Mesaconine | Y=1.22384e5x+10811.41645 | 0.99083 | 0.21-600 | 0.02 | 0.06 |
| Hypaconine | Y=3.72337e5x+5936.54051 | 0.99200 | 0.82-391 | 0.01 | 0.03 |
| Hypaconitine | Y=7.45254e5x+19296.70962 | 0.99642 | 0.35-1000 | 0.01 | 0.03 |
| Mesaconitine | Y=3.47687e5x+1686.24234 | 0.99720 | 0.26-1500 | 0.07 | 0.21 |
| Lappaconitine | Y=16506.19222x+5995.21516 | 0.99522 | 0.43-3125 | 0.15 | 0.43 |
| Benzoylaconine | Y=4.89846e5x+12564.70768 | 0.99851 | 19.79-2300 | 0.01 | 0.03 |
| Benzoylmesaconine | Y=1.43086e5x+9.20415e4 | 0.99056 | 0.76-1200 | 0.03 | 0.08 |
| Benzoylhypaconine | Y=4.89846e5x+12564.70678 | 0.99719 | 0.35-333 | 0.04 | 0.12 |
| Karacoline | Y=5.26083e4x+8574.04708 | 0.99755 | 0.76-5500 | 0.04 | 0.08 |
| Fuziline | Y=6.38638e4x+3.20814e4 | 0.99641 | 0.56-4000 | 0.02 | 0.06 |
| Songorine | Y=1.98696e5x+688.38233 | 0.99122 | 0.87-1500 | 0.03 | 0.09 |

**Table S5 The recovery and stability of 12 components**

| **Compound** | **Repeatability**  **(RSD, %, n=6)** | **Intra-day precision**  **(RSD, %, n=6)** | **Inter-day precision**  **(RSD, %, n=6)** |
| --- | --- | --- | --- |
| Aconine | 4.80±0.76 | 4.35±0.92 | 2.66±0.29 |
| Mesaconine | 8.09±0.31 | 4.99±0.34 | 3.90±0.14 |
| Hypaconine | 1.09±0.47 | 5.05±0.32 | 1.67±0.21 |
| Hypaconitine | 9.15±0.94 | 8.01±0.80 | 8.18±0.65 |
| Mesaconitine | 2.22±0.04 | 2.82±0.02 | 7.50±0.64 |
| Lappaconitine | 7.01±0.02 | 8.69±0.83 | 3.12±0.97 |
| Benzoylaconine | 7.14±0.22 | 4.86±0.10 | 4.59±0.71 |
| Benzoylmesaconine | 2.10±0.38 | 5.95±0.45 | 4.57±0.72 |
| Benzoylhypaconine | 8.93±0.77 | 5.52±0.59 | 2.61±0.07 |
| Karacoline | 3.66±0.21 | 9.78±0.27 | 2.24±0.84 |
| Fuziline | 6.49±0.88 | 8.88±0.20 | 5.37±0.83 |
| Songorine | 9.40±0.27 | 6.36±0.14 | 8.62±0.65 |

**Table S6 The recovery and stability of 12 components**

| **Compound** | **Content (%)** | **Average recovery (%, n=6)** | **RSD**  **(%, n=6)** | **Stability (RSD, %,n=7)** |
| --- | --- | --- | --- | --- |
| **Aconine** | 1.22±0.10 | 97.20±0.94 | 2.98±0.70 | 5.35±0.25 |
| **Mesaconine** | 3.43±0.83 | 98.27±0.23 | 1.61±0.14 | 4.12±0.97 |
| **Hypaconine** | 3.10±0.73 | 96.24±0.17 | 1.52±0.13 | 3.52±0.26 |
| **Hypaconitine** | 7.84±0.63 | 98.33±0.22 | 4.10±0.98 | 6.17±0.36 |
| **Mesaconitine** | 3.31±0.33 | 98.69±0.60 | 2.47±0.09 | 8.23±0.20 |
| **Lappaconitine** | 8.13±0.32 | 92.64±0.47 | 6.02±0.27 | 1.51±0.14 |
| **Benzoylaconine** | 4.99±0.65 | 97.70±0.45 | 9.45±0.14 | 3.37±0.91 |
| **Benzoylmesaconine** | 9.25±0.87 | 103.37±0.30 | 9.25±0.34 | 2.90±0.58 |
| **Benzoylhypaconine** | 7.76±0.38 | 98.01±0.61 | 6.59±0.99 | 9.73±0.41 |
| **Karacoline** | 4.86±0.52 | 101.50±0.57 | 2.31±0.95 | 3.86±0.74 |
| **Fuziline** | 7.03±0.57 | 98.68±0.64 | 4.90±0.05 | 7.97±0.67 |
| **Songorine** | 2.04±0.16 | 101.71±0.27 | 5.87±0.20 | 9.90±0.65 |
